# Uptake of Risk‐Reducing Salpingo‐Oophorectomy and Gynaecologic Surveillance Among Germline *BRCA* Pathogenic Variants Carriers

**DOI:** 10.1002/cam4.70321

**Published:** 2024-12-03

**Authors:** Alessandra Inzoli, Serena Negri, Cristina Dell'Oro, Clarissa Costa, Liliana Marchetta, Mariaclara Boccadutri, Simona Fumagalli, Gaia Roversi, Elena Maria Sala, Chiara Celi, Valentina Rossi, Robert Fruscio

**Affiliations:** ^1^ Department of Medicine and Surgery University of Milan‐Bicocca Milan Italy; ^2^ Medical Genetics Fondazione IRCCS San Gerardo dei Tintori Monza Italy; ^3^ Clinical Psychology Unit IRCCS Fondazione San Gerardo dei Tintori Monza Italy; ^4^ Gynecology Unit IRCCS Fondazione San Gerardo dei Tintori Monza Italy

**Keywords:** *BRCA* mutation, cancer prevention, psychosocial studies, women's cancer

## Abstract

**Introduction:**

Risk‐reducing salpingo‐oophorectomy (RRSO) is recommended by international guidelines in women with *BRCA1/2* germline pathogenic variants (PV) to prevent ovarian cancer. Despite the solid recommendation, women frequently refuse surgery and uptake rates reported in the literature are diverse. This study analyses the uptake rate of RRSO in BRCA 1/2 PV‐carriers referred to a specialised referral centre for first counselling and investigate personal factors linked to the decision.

**Methods:**

This is a single‐centre prospective study of *BRCA1/2* PV‐carriers referred for the first counselling to IRCCS Fondazione San Gerardo de’ Tintori (Monza, Italy) between January 2010 and May 2023. Depending on individual characteristics, women were either proposed RRSO or surveillance, consisting of transvaginal ultrasound and CA125 measurement twice per year according to Regional guidelines. Women within the centre have access to a clinical psychologist, a nutritional consult and treatment of menopausal atrophy with diode vaginal laser. The primary endpoint of the study was the uptake rate of RRSO. The secondary objective was to evaluate the main reasons for refusing surgery.

**Results:**

Among the 287 women included, surgery was proposed to 205 women either at first counselling or during surveillance and was accepted by 197, with an uptake rate of 96.1%. 17.25% of women met the psychologist before or after surgery. The main reasons for refusing RRSO were fear of iatrogenic menopause and childbearing desire.

**Conclusion:**

This study shows a high uptake rate of RRSO in *BRCA* PV‐carriers. We believe that the presence of a dedicated outpatient clinic with a multidisciplinary team contributes decisively to our results. Gynaecologic surveillance, as though not beneficial in terms of oncological prevention, may play a significant role in encouraging women with *BRCA* PV to opt for risk‐reducing surgery.

## 1 Introduction

Ovarian cancer (OC) is the third most common gynaecologic cancer, with an estimated incidence of 314,000 cases yearly [1]. Although it is not the most common ones, OC is the leading cause of mortality from gynaecologic malignancy [2] due to its poor prognosis when diagnosed late. In fact, 80% of patients with OC are diagnosed with advanced disease, and the overall survival after 5 years is 47% (89% for I‐stage disease, 20% for IV‐stage disease) [3].

In Italy, 6000 estimated new diagnosis of ovarian cancer were made in 2022, with an overall survival after 5 years of 43% [4].

Women with germline *BRCA1* or *BRCA2* pathogenic variants (PV) account for 5% of all breast cancer (BC) and 15% of all OC [5, 6]. Women with *BRCA1* PV have a lifetime risk of developing OC of 31%–59%, while for *BRCA2* PV‐carriers the risk is between 6% and 34% [7].

Given the high mortality rate of OC, prevention of new cases is crucial. Prophylactic surgery with risk‐reducing salpingo‐oophorectomy (RRSO) remains the only scientifically proven strategy to reduce the risk of ovarian and tubal cancer [8], with proved efficacy than intensive screening and chemoprevention [9, 10]. Indeed, serum CA125 measurement and transvaginal ultrasound performed routinely do not reduce the risk or the mortality of OC, and are unreliable for early diagnosis [11]. Oral contraceptive use has been associated with reduced risk of OC, even after cessation use [12]. However, the potential and controversial increased risk of BC and venous thromboembolism among users has not resulted in a universal recommendation [13].

RRSO has been shown to decrease the probability of OC by 96% [14] and all causes mortality by 76% [15] in women with *BRCA* PV.

The National Comprehensive Cancer Network (NCCN) suggests that women with a germline BRCA1 pathogenic variant should consider undergoing RRSO between the ages of 35 and 40 or after completing childbearing [16]. For women with BRCA2 pathogenic variants, it may be reasonable to delay the surgery until they are between 40 and 45 years old, given the later onset of ovarian cancer [17].

Although there is a solid recommendation for undergoing RRSO, women are frequently reluctant to perform surgery. Previous studies have shown that there are many differences, in terms of uptake rates, between countries and hospitals, ranging between 15% and 78% [18, 19, 20, 21, 22, 23, 24]. Premenopausal women who undergo RRSO experience premature menopause and beyond losing their fertility, they experience a significant and sudden worsening of their quality of life, as menopausal symptoms, such as hot flashes, mood swings, sleep disorders and sexual dysfunction, arise right after surgery. Also, premature menopause increases the risk of losing bone mass [25] and of long‐term sequelae such as cardiovascular diseases, cognitive impairment, dementia, anxiety and depression [26]. It has been demonstrated that hormonal replacement therapy (HRT) is able to balance these negative effects, however many women with *BRCA1/2* PV have a previous history of BC, which hampers the possibility to assume HRT.

A recent study [27] highlighted the role of counselling in decision‐making for women at high inherited risk of OC to improve the outcomes. One of the principal matters is to increase the uptake of RRSO, to improve the survival of *BRCA1/2* PV‐carriers.

The present study analyses the uptake rate of prophylactic surgery in women with pathogenic germline *BRCA1/2* mutations followed in a dedicated and multidisciplinary outpatient clinic. The secondary aim is to investigate personal factors that influence the decision to either have the surgery or decline it.

## 2 Materials and Methods

This is a single‐centre prospective study of all patients who were diagnosed with a *BRCA1/2* germline PV and were referred for the first counselling to IRCCS Fondazione San Gerardo de’ Tintori (Monza, Italy) between January 2010 and May 2023. Women were excluded from the analysis if they (i) already underwent bilateral salpingo‐oophorectomy for other reasons at the time of diagnosis, (ii) performed RRSO in other centres or (iii) received their first counselling visit at other centres. The primary endpoint was the acceptance rate of RRSO. The secondary objective was to evaluate the main reasons for refusing surgery.

The positive result of genetic testing is communicated to women in presence of a geneticist, a gynaecologist and a senologist. In this occasion, general information, tailored on the age and health status of women, are provided. All women have the possibility to have an appointment in a gynaecologic outpatient clinic dedicated to women at high inherited risk of gynaecological malignancies, which is provided within 1 month from the result. The proposal of the preventive strategy is tailored on age, previous medical history and desire of pregnancies. According to the guidelines of the Lombardy region [28] screening with transvaginal ultrasound, gynaecological examination and measurement of CA125 twice a year starting from the age of 30 years is recommended. RRSO is proposed at the latest at 40 years (for *BRCA1* PV‐carriers) or 45 years (for *BRCA2* PV‐carriers), as recommended by many international guidelines [16]. The Regional Health System covers all the costs of these examinations and of the prophylactic surgery. Women who perform genetic testing after the recommended age for surgery are directly proposed prophylactic surgery. During counselling, women are informed about the recommendation to perform RRSO, its surgical risks and the indications for HRT. Surgery includes bilateral salpingo‐oophorectomy, peritoneal washing and endometrial sampling. We explicitly inform women that screening for OC is not effective for early diagnosis and cannot replace surgery. At the outpatient clinic a clinical psychologist is available for all women who need support in dealing with the diagnosis of the mutational status or with the acceptance of the intervention. Finally, a nutritional consult with a dedicated clinical nutritionist is offered to women that are afraid of the metabolic consequences of iatrogenic menopause. Elements of counselling are shown in Table S1 [25, 26, 29, 30, 31, 32, 33, 34, 35, 36, 37, 38, 39, 40, 41].

For all women, clinical data (age, parity, desire for childbearing, menopausal state), pathological anamnesis (personal history of breast cancer and personal history of risk‐reducing mastectomy), family history data (cases of cancer in first‐degree relatives, cases of death by cancer in first‐degree relatives and relatives in general) and genetic data (type of the PV) were collected.

This study was approved by the local IRB (Comitato Etico “Brianza”) and performed by the ethical standards in the Declaration of Helsinki. Informed consent was obtained from all individual participants.

## 3 Results

A total of 335 women were eligible for the study, and 48 were excluded according to exclusion criteria: 29 women performed RRSO at other centres, 11 women already underwent surgery for other reasons, and 8 women received first counselling visits at other centres. Among the 287 women included, 157 had *BRCA1* PV, 128 had *BRCA2* PV and 2 women had both *BRCA1* and *BRCA2* PV. Table 1 shows the women’ characteristics at the first counselling visit. The mean age at genetic testing was 43 years for both *BRCA1/2* PV‐carriers. 53.4% of women with *BRCA1* PV and 40% of women with *BRCA2* PV received the results of the genetic testing and the first counselling visit before the recommended age for surgery. The mean age at surgery for *BRCA1* PV‐carriers was 46 years old, while for *BRCA2* PV‐carriers was 52 years old. Most women (64.1%) with *BRCA1* PV were premenopausal at diagnosis, while 52% of women with *BRCA2* PV were postmenopausal. The majority of women had no childbearing desire at the moment of the diagnosis (61.6% of *BRCA*1 and 79.6% of *BRCA2* PV‐carriers). The most common cancer in relatives was BC (36.4% for *BRCA1* and 44.5% for *BRCA2* PV‐carriers), which was also the first cause of death from cancer in relatives (24.5% and 21%); however, this data was missing for around 20% of women. Nearly half of the women had a personal history of BC (42.7% for *BRCA1* and 50% for *BRCA2*).

**TABLE 1:** Baseline women's characteristics.

Women's characteristics (*n* = 287) mean ± SD (range or %)	*BRCA1* ^a^ (*n* = 159)	*BRCA2* (*n* = 128)
Age at diagnosis (years) range	43 ± 12 (18–80)	43 ± 12 (22–77)
Diagnosis within the recommended age for surgery	85 (53.4%)	52 (40%)
Age at surgery^b^ (years) range	46.8 ± 7.4 (32–66)	52.5 ± 8.3 (34–78)
Reason for genetic testing		
Familiarity	99 (62.2%)	71 (55.5%)
Personal history of breast cancer	60 (37.8%)	57 (44.5%)
Menopausal status at diagnosis		
Premenopausal	102 (64.1%)	47 (36.7%)
Postmenopausal	43 (27%)	67 (52%)
Current use of hormonal therapy for breast cancer	13 (8.1%)	14 (10.9%)
Parity		
0	69 (43.3%)	40 (31.2%)
1	36 (22.6%)	40 (31.2%)
2	39 (24.5%)	40 (31.2%)
> 2	14 (8.8%)	8 (0.6%)
Unknown	1 (0.6%)	—
Childbearing desire		
Yes	54 (33.9%)	25 (19.5%)
No	98 (61.6%)	102 (79.6%)
Unknown	7 (4.4%)	1 (0.7%)
Cases of cancer in I‐degree relatives		
None	29 (18%)	25 (19%)
Breast cancer	58 (36.4%)	57 (44.5%)
Ovarian cancer	29 (18%)	28 (21.8%)
Breast cancer and ovarian cancer	42 (26.4%)	16 (12.5%)
Unknown	1 (0.6%)	2 (1.5%)
Cases of death by cancer in I‐degree relatives		
None	94 (59%)	67 (52.3%)
Breast cancer	15 (9.4%)	17 (13.2%)
Ovarian cancer	13 (8%)	8 (6.2%)
Breast cancer and ovarian cancer	1 (0.6%)	1 (0.7%)
Unknown	36 (22.6%)	35 (27.3%)
Personal history of breast cancer	68 (42.7%)	64 (50%)
Previous risk‐reducing mastectomy	14 (0.8%)	4 (3.1%)

^a^ Two patients with *BRCA1* and *BRCA2* mutation.

^b^ Missing data for three patients who underwent surgery at other centres after follow‐up.

**FIGURE 1:**
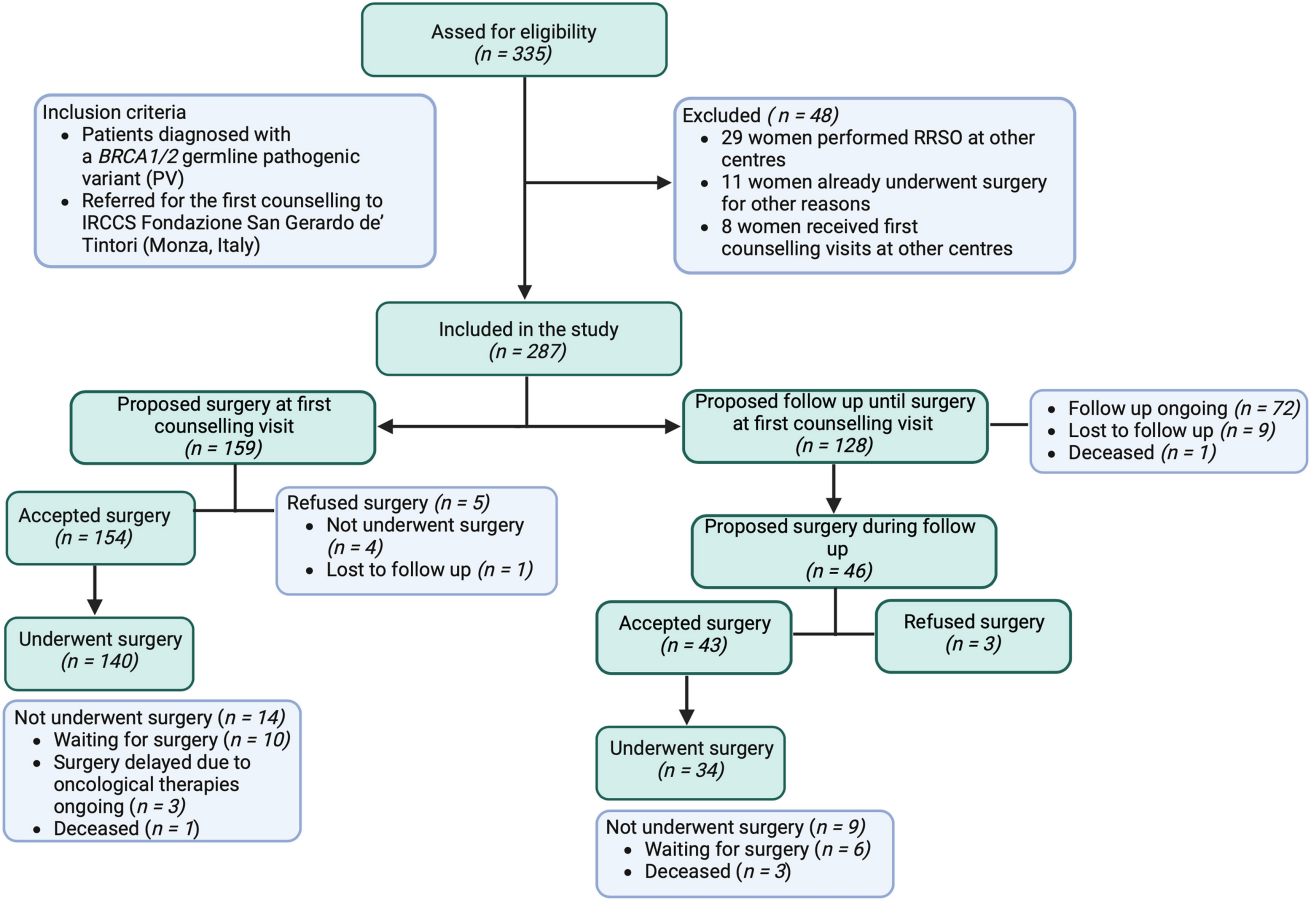
Flowchart of study.

Flowchart of study is shown in Figure 1.

Surgery was proposed at first counselling to 159 women; their characteristics are shown in Table S2. One hundred and fifty‐four women accepted (96.8%), while only five (3.2%) declined. Of these five, one was lost at follow‐up and four are still on follow‐up. The median interval between proposal and surgery was 5 months (range 1–20 months). Eleven women who accepted the proposal (7.1%) met the psychologist before surgery, and in particular two of them who initially declined surgery ended up accepting the proposal after meeting with the psychologist. Eleven women met the psychologist after surgery (7.1%).

Surveillance until surgery was proposed to 128 women. During follow‐up, surgery was eventually proposed to 46 of them; their characteristics are shown in Table S3. Forty‐three women accepted (93.4%) while three declined (6.4%) and are still undergoing surveillance. Among the ones who accepted surgery, 10 (23.3%) met the psychologist before surgery and 2 (4.7%) after surgery. The median interval between proposal during follow‐up and surgery was 6.5 months (range 1–23).

Among the remaining 82 women, surgery has not been proposed yet to 72, while 9 are lost at follow‐up and 1 died due to breast cancer progression during follow‐up.

Totally, surgery was proposed to 205 women and was accepted by 197 (96.1%). The characteristics of these women are listed in Table 2. Women accepted surgery after adequate counselling, since they understood the beneficial role of RRSO as the only way to prevent OC.

**TABLE 2:** Characteristics of women according to the acceptance of surgery (proposed at first visit or during follow‐up).

Women's characteristics (*n* = 205) mean ± SD (range or %)	Surgery proposed at first visit or during follow‐up (*n* = 205)	
	Accepted (*n* = 197)	Declined (*n* = 8)
Mutational status		
*BRCA1* ^a^	97 (49.2%)	7 (87.5%)
*BRCA2*	100 (50.8%)	1 (12.5%)
Age at diagnosis	48.5 ± 9.1 (23–77)	45.1 ± 11.3 (35–72)
Reason for genetic testing		
Familiarity	98 (49.7%)	3 (37.5%)
Personal history of breast cancer	99 (50.3%)	5 (62.5%)
Psychological interview		
No	163 (82.7%)	6 (75%)
Yes	34 (17.3%)	2 (25%)
Menopausal status		
Premenopausal	73 (37%)	7 (87,5%)
Postmenopausal	106 (53,8%)	1 (12,5%)
Current use of hormonal therapy for breast cancer	17 (10,8%)	—
Previous hysterectomy for benign reason	1 (0,5%)	—
Parity		
0	40 (20.3%)	4 (40%)
1	65 (32.9%)	2 (30%)
2	72 (36.6%)	2 (30%)
> 2	19 (9.7%)	0 (0%)
Unknown	1 (0.5%)	
Childbearing desire		
Yes	15 (7.6%)	4 (50%)
No	177 (89.8%)	4 (50%)
Unknown	5 (2.6%)	—
Cases of cancer in I degree relatives		
None	38 (19.5%)	2 (20%)
Breast cancer	78 (40%)	4 (40%)
Ovarian cancer	41 (21%)	2 (20%)
Breast cancer and ovarian cancer	35 (17.9%)	0 (0%)
Unknown	3 (1.6%)	—
Cases of death by cancer in I degree relatives		
None	102 (51.8%)	3 (37.5%)
Breast cancer	25 (12.7%)	3 (37.5%)
Ovarian cancer	18 (9.1%)	—
Breast cancer and ovarian cancer	1 (0.5%)	—
Unknown	51 (25.9%)	2 (25%)
Personal history of breast cancer	112 (56.8%)	5 (62.5%)
Risk‐reducing mastectomy in personal medical history	13 (6.5%)	1 (12.5%)

^a^ Two patients with *BRCA1* and *BRCA2* mutation.

Among patients who declined surgery, seven had a *BRCA1* PV, and one had a *BRCA2* PV; seven were premenopausal, and five had a personal history of BC. Mean age at diagnosis was 48 in accepted group versus 45 in declined group, with a prevalence of premenopausal status of 87% in those who declined versus 37% in those who accepted. Of the 82 women with a I degree relative with history of breast cancer, 95.1% of them accepted surgery. The uptake rate was high also for patients with I degree relatives with ovarian cancer (95.3%), and among women with both breast and ovarian cancer in I degree relatives the uptake rate was 100%.

Totally, 161 patients underwent RRSO. Of them 149 had negative results, 5 had occult cancer, 6 had serous tubal intraepithelial carcinoma (STIC) and 1 had serous tubal intraepithelial lesion (STIL) at the histological report.

The reasons for refusing RRSO are shown in Table 3 and were fear of consequences of iatrogenic menopause (*n* = 4), childbearing desire (*n* = 2), fear of surgery (*n* = 1) and unknown (*n* = 1). Among the four women who declined surgery because of the consequences of iatrogenic menopause, three (Patients 1, 4 and 6) had contraindications to HRT since they had BC and the other one (Patient 5) was afraid of its psychological impact. She is currently meeting with the psychologist in order to try to accept the proposal. One woman (Patient 4) decided to have bilateral salpingectomy at another centre. Two women declined surgery for childbearing desire and one of them had a pregnancy. One woman (Patient 7) declined surgery for comorbidities and age (72 years old). For the last one the reason for refusing surgery is unknown and she is lost at follow‐up.

**TABLE 3:** Characteristics of women who declined surgery.

Ref	Age at proposal	Mutation	Menopausal state	Personal history of BC	Reason for refusal	Timing of refusal
1	42	BRCA1	Premenopausal	Yes	Afraid of iatrogenic menopause	First visit
2	41	BRCA1	Premenopausal	Yes	Afraid of iatrogenic menopause	First visit
3	46	BRCA1	Premenopausal	No	Afraid of iatrogenic menopause	First visit
4	43	BRCA1	Premenopausal	Yes	Afraid of iatrogenic menopause	During follow‐up
5	39	BRCA1	Premenopausal	No	Childbearing desire	During follow‐up
6	40	BRCA1	Premenopausal	No	Childbearing desire	During follow‐up
7	72	BRCA2	Postmenopausal	Yes	Fear of surgery	First visit
8	43	BRCA1	Premenopausal	Yes	Unknown	First visit

## 4 Discussion

Risk‐reducing salpingo‐oophorectomy is the effective strategy to prevent ovarian cancer in high‐risk patients and is therefore recommended by international guidelines. However, the uptake rate of surgery is reported to be very variable [18, 19, 20, 21]. Understanding factors implied in the decision‐making of women may help improving the acceptability of the intervention and ultimately their survival.

Our study shows an uptake rate of 96.1% in *BRCA1‐2* PV carriers referred for first counselling to a specialised centre that focuses on individuals with genetic susceptibility to ovarian cancer, which is higher than the rates previously reported in the literature [21].

Skytte et al. [40] report an uptake rate of 75% at 10 years in unaffected *BRCA*‐PV carriers who perform annual transvaginal ultrasound and serum CA125 from age 30, according to national guidelines. Among the reasons for this high uptake rate, the authors enlist free access to surveillance and surgery within the Health Care System and a multidisciplinary approach with regular clinical discussion about the management of *BRCA* families. The uptake rate is also high in the study of Madalinska et al. [19], with 74% at 12 months, and insurance coverage was cited as a factor that could influence patients' decisions. Indeed, the access to a free Health Care System or the availability of an insurance coverage for the surgery may play an important factor and explain the differences in uptake rates between countries. In Italy, both gynaecologic surveillance and risk‐reducing salpingo‐oophorectomy are covered by the National Health Care System, and this could also contribute to the high uptake rate observed.

*BRCA1* PV carriers account for 87.5% of refusals. Literature reports on *BRCA1‐2* PV prevalence of patients who refuse surgery have conflicting results [41, 42]. Given that patients with *BRCA1* PV mutations must undergo surgery before those with *BRCA2*, it is not surprising that they are the ones who refuse surgery the most.

Patients who declined surgery were slightly younger than those undergoing surgery, with a mean age of 45 and 48 respectively, a finding consistent with published results [43]. As Bradbury et al. [44] already reported, the desire for children is one of the leading reasons for refusing surgery. Indeed, in our study, 87.5% of those who declined surgery were premenopausal, and 28.6% expressed a desire to conceive. It is important to note that the uptake rate among patients without previous pregnancies in our study is 90.9%, which is significantly higher than the reported rate of 30% in the literature [40, 44].

Family history of breast or ovarian cancer may play a decisive role in the decision to undergo risk‐reducing surgery. In the general population, women with a strong family history for BC are more likely to have medical preventive behaviour than women who do not have positive family history [45]. Likewise in the general population, uptake rates for prophylactic surgery are reported to be higher in *BRCA1/2* PV‐carriers with a mother or a sister with OC [43]. In our study, BC was the most common cancer in first degree relatives, and the leading cause of death in first degree relatives since 11% of patients had a mother or a sister or a daughter who died due to BC. However, in our population family history does not seem to play a role in the acceptance of preventive surgery. In fact, among women with a negative family history the uptake rate was 95% (38/40), and it was not different compared to women with first degree relatives with BC (95%, 78/82) or OC (95%, 41/43) or both (100%, 35/35).

A personal history of BC is often reported as the main reason for accepting surgery [20, 21]. However, in this cohort, 56.8% of the patients who accepted the intervention and 62.5% of those who declined it had a personal history of BC. One of the reasons could be that even if women with a history of BC are generally more worried about having another cancer, they cannot receive hormone replacement therapy (HRT), making them more susceptible to and afraid of surgical menopause.

The fear of iatrogenic menopause was the most common reason for refusing the risk‐reducing surgery (50% of refusals). After undergoing RRSO, women experiencing surgical menopause tend to suffer from more frequent vasomotor symptoms, trouble sleeping, feelings of depression and tiredness, and sexual dysfunctions as compared to natural menopause [33]. Vasomotor symptoms arise gradually over a few months during natural menopause, and in 80%–90% of cases, they resolve in 4–5 years [34]. However, in surgical menopause, they are suddenly abrupt and may be more severe, frequent and long‐lasting [35]. Almost all these symptoms are unavoidable in the majority of cases; in fact, many women with *BRCA1/2* PV cannot take HRT, for a previous history of breast cancer. However, we tried to minimise their impact on the decision to accept surgery and on the quality of life offering complimentary services, such as psychological support. Also the consultation with a clinical nutritionist, who explains how to modify their lifestyle and eating habits in order to contain menopausal symptoms [36], including vasomotor symptoms, changes in bodyweight and composition, psychological symptoms [37, 38], sleep disturbances and urogenital symptoms, has been appreciated by many of our women. Another recent addition has been offering the diode vaginal laser for the treatment of menopausal atrophia; the first results of this strategy are encouraging [39]. Finally, an effective strategy to postpone premature menopause while still preventing ovarian cancer could be salpingectomy with delayed oophorectomy. At our institution women can be enrolled in the TUBA‐WISP II study [46] (NCT04294927), and they can choose between bilateral salpingectomy with delayed oophorectomy and the traditional bilateral salpingo‐oophorectomy. Delaying surgical menopause for at least 5 years could have a significant impact on quality of life and long term health, especially in *BRCA1* PV carriers with a personal history of breast cancer. From our study, 14 patients were included in the TUBA WISP II study and accepted. Of them, 11 have undergone surgery, three were waiting for surgery at the time of inclusion.

Our study suggests a possible importance of the role of the clinical psychologist in the management of *BRCA* PV carriers. Genetic testing can have a life‐changing impact on women who test positive for a high risk of developing cancer, causing adverse psychological effects such as anger, sadness or guilt [47]. For most women, the genetic test coincides with a personal diagnosis of cancer or a similar diagnosis in a close relative. Moreover, prophylactic salpingo‐oophorectomy is linked to an increased risk of body image distress [48], and we already reported that the intervention of a clinical psychologist can improve the quality of life after prophylactic adnexectomy [49]. The effectiveness of a multidisciplinary strategy is confirmed by other studies [50, 51, 52]. Metcalfe et al. [50] designed a randomised study to assess the role of a theory‐based behavioural intervention delivered by genetic counsellors in the uptake rates of women with a *BRCA* PV. Patients were randomised to a follow‐up genetic counselling by telephone based on theoretical constructs aimed at motivating individuals with *BRCA* PV to undergo prophylactic RRSO. Although after 1 year the uptake rates between the two groups remained the same, the group of patients randomised to the intervention showed lower levels of decisional conflict and higher knowledge levels than those who received standard care. At 2 years, the uptake rates in the intervention group were higher with statistical significance. This highlights the role of constant counselling in increasing the uptake rates of women with *BRCA* PV.

Finally, we are aware that surveillance with transvaginal ultrasound and CA125 is not effective in reducing mortality for OC both in the general [53] and in the high risk population [54]. However, the possibility for women to meet a multidisciplinary team twice a year allows them to understand the importance of the intervention and to ask questions, to express concerns and fears that can be faced and resolved.

The strengths of our study are that we evaluated all patients referred to our institution, therefore reducing selection bias, and that all patients performed their first counselling visit with the same physician. A possible limitation is the older age of our patients compared to other studies, which can influence the high uptake rate.

In conclusion, our study shows a high uptake rate of RRSO in *BRCA1/2* PV‐carriers referred to our Institution for first counselling and subsequent follow‐up. We believe that our results are mainly a consequence of the multidisciplinary team involved in managing these women working together in a dedicated outpatient clinic. Gynaecologic surveillance, as though not beneficial in terms of oncological prevention, may play a significant role in encouraging patients with *BRCA1‐2* PV to opt for risk‐reducing surgery.

## Author Contributions

**Alessandra Inzoli:** data curation (equal), formal analysis (equal), funding acquisition (equal), investigation (equal), supervision (equal), writing – original draft (equal), writing – review and editing (equal). **Serena Negri:** data curation (equal), supervision (equal), writing – original draft (equal), writing – review and editing (equal). **Cristina Dell'Oro:** data curation (equal), formal analysis (equal). **Clarissa Costa:** data curation (equal), formal analysis (equal). **Liliana Marchetta:** data curation (equal), formal analysis (equal). **Mariaclara Boccadutri:** data curation (equal), formal analysis (equal). **Simona Fumagalli:** conceptualization (equal), writing – review and editing (equal). **Gaia Roversi:** conceptualization (equal), writing – review and editing (equal). **Elena Maria Sala:** conceptualization (equal), writing – review and editing (equal). **Chiara Celi:** conceptualization (equal), writing – review and editing (equal). **Valentina Rossi:** conceptualization (equal), writing – review and editing (equal). **Robert Fruscio:** conceptualization (lead), data curation (equal), investigation (lead), project administration (equal), supervision (lead), writing – original draft (equal), writing – review and editing (lead).

## Ethics Statement

This study was approved by the local IRB (Comitato Etico “Brianza”) and performed in accordance with the ethical standards in the Declaration of Helsinki. Informed consent was obtained from all individual participants included in the study.

## Conflicts of Interest

The authors declare no conflicts of interest.

## Supporting information


Table S1.

## Data Availability

The datasets generated during and/or analyzed during the current study are available from the corresponding author on reasonable request.
