# Supplementary material for: Uptake of Risk‐Reducing Salpingo‐Oophorectomy and Gynaecologic Surveillance Among Germline BRCA Pathogenic Variants Carriers
Source: Cancer Med. 2024 Dec 3;13(23):e70321. doi: 10.1002/cam4.70321 (PMC11612664; doi:10.1002/cam4.70321)
Supplement: Supplementary file 1 — Table S1. [file CAM4-13-e70321-s001.docx]

**Appendix**

**Supplementary Table 1**

Elements of counseling

|  | **Communicating the risk** | **Preventive / treatment options** | **Ref** |
| --- | --- | --- | --- |
| **Carrier of PV BRCA 1** | *Your risk of developing ovarian cancer is 39-59%, the one for the general population is 1.1%.* | *The only proven strategy to reduce the risk of ovarian cancer is RRSO which reduces the risk of developing it of 85-90%. According to Guidelines, you should undergo RRSO between 35-40 years old.* | [16][29][30]  [31][32] |
|  | *Your risk of developing breast cancer is 57-72%, while for general population is 12.9%.* | *According to Guidelines, to reduce the risk of breast cancer you can perform risk-reducing prophylactic mastectomy which decreases the risk of breast cancer by 90%. However, the choice is based on your preference, given that effective screening is available.* | [16][31][33] |
| **Carrier of PV BRCA 2** | *Your risk of developing ovarian cancer is 11-20%, the one for the general population is 1.1%.* | *The only proven strategy to reduce the risk of ovarian cancer is RRSO which reduces the risk of developing it of 85-90%. According to Guidelines, you should undergo RRSO between 40-45 years old.* | [16][29][30]  [31][32] |
|  | *Your risk of developing breast cancer is 45-69%%, while for general population is 12.9%.* | *According to Guidelines, to reduce the risk of breast cancer you can perform risk-reducing prophylactic mastectomy which decreases the risk of breast cancer by 90%. However, the choice is based on your preference, given that effective screening is available.* | [16][31][33] |
| **Patient on fertile age at the time of RRSO, without history of breast cancer** | *The main consequence of RRSO is surgical and premature menopause, whose symptoms can impair your quality of life.* | *Luckily, you have no contraindications to hormonal replacement therapy (HRT), so you will be prescribed HRT which will ameliorate all the symptoms of menopause and will prevent long-term consequences such as osteoporosis and increased risk of cardiovascular disease. This will not increase your risk of developing breast cancer if we stop HRT between 45-50 years old. At our institution, we have a psychologist who can help you with the acceptance of the surgery. If you want, we also have a nutritionist which can help you with metabolic consequences of iatrogenic menopause.* | [25][26][30]  [35][36][37]  [38][39][40]  [41] |
| **Patient on fertile age at the time of RRSO, with a history of breast cancer** | *The main consequence of RRSO is surgical and premature menopause, whose symptoms can impair your quality of life.* | *Unfortunately, since you have a history of breast cancer, you have an absolute contraindication to the use of HRT. However, there are some non-hormonal drugs which can be used for the management of the symptoms of menopause. In besides, at our institution we have laser-therapy for the treatment of genitourinary syndrome of menopause which is useful for women with contraindications to HRT. If you wish, we have a psychologist who can help you with the acceptance of the surgery and to deal with consequences of surgical menopause. If you want, we also have a nutritionist which can help you with metabolic consequences of iatrogenic menopause.* | [25][26][35]  [36][37][38]  [39] |
| **Menopausal patient at the time of RRSO** | *The main consequence of RRSO is surgical and premature menopause, whose symptoms can impair your quality of life. However, since you are already on menopause the impact will be much lower.* | *At our institution, we have a psychologist who can help you with the acceptance of the surgery.* | [25][26][30] |

39

**Supplementary Table 2**

Characteristics of women with surgery proposal at baseline

| **Women’s characteristics (n= 159)**  *Mean ± SD (range or %)* | **Surgery proposal at baseline**  **(n = 159)** | |
| --- | --- | --- |
|  | **Accepted**  **(n = 154)** | **Refused**  **(n = 5)** |
| Mutational status   - BRCA1 - BRCA2 | 72 (46,7%)  82 (53,3%) | 4 (80 %)  1 (20 %) |
| Age at diagnosis | 50,7 ± 8  (36 - 73) | 51,7±7,5  (40 - 72) |
| Reason for genetic testing   - Familiarity - Personal history of breast cancer | 76 (49,4%)  78 (50,6%) | 1 (20%)  4 (80%) |
| Menopausal status   - premenopausal - postmenopausal - current use of hormonal therapy for BC - previous hysterectomy for benign reasons | 49 (31,9 %)  94 (61 %)  10 (6,5 %)  1 (0,6 %) | 4 (80 %)  1 (20 %)  -  - |
| Parity   - 0 - 1 - 2 - > 2 - unknown | 27 (17,5 %)  51 (33,1 %)  61 (39,6 %)  14 (9,2 %)  1 (0,6 %) | 4 (80 %)  -  1 (20 %)  -  - |
| Childbearing desire   - yes - not - unknown | 2 (1,3 %)  149 (96,8 %)  3 (1,9 %) | 1 (20 %)  4 (80 %)  - |
| Phsycological interview   - no - yes | 132 (85,8%)  22 (14,2%) | 3 (60%)  2 (40%) |
| Months between proposal and surgery^+^ | 5,3 ± 3,9  (1-20) | - |
| Cases of cancer in I degree relatives   - none - breast cancer - ovarian cancer - breast cancer and ovarian cancer - unknown | 29 (19%)  63 (40,9%)  34 (22%)  26 (16,8%)  2 (1,3%) | -  4 (80%)  -  1 (20%)  - |
| Cases of death by cancer in I degree relatives   - none - breast cancer - ovarian cancer - breast cancer and ovarian cancer - unknown | 76 (49,4%)  19 (12,4%)  14 (9%)  1 (0,6%)  44 (28,6 %) | 2 (40 %)  3 (60 %)  -  -  - |
| Personal history of breast cancer | 91 (59%) | 4 (80 %) |
| Previous risk-reducing mastectomy | 11 (7,1 %) | 1 (20%) |

**Supplementary Table 3**

Characteristics of women with surgery proposal during follow-up

| **Women’s characteristics (n= 46)**  *Mean ± SD (range or %)* | **Surgery proposal during follow up**  **(n = 46)** | |
| --- | --- | --- |
|  | **Accepted**  **(n = 43)** | **Refused**  **(n = 3)** |
| Mutational status   - BRCA1† - BRCA2 | 25 (58,1 %)  18 (41,8 %) | 3 (100%)  0 (0%) |
| Age at diagnosis | 43,8 ± 10  (23 - 77) | 40,5 ± 3,43  (35-43) |
| Reason for genetic testing   - Familiarity - Personal history of breast cancer | 22 (51,2%)  21 (48,8%) | 1 (33,3%)  2 (66,7%) |
| Months of follow up | 23,6 ± 15  (5-62) | 14 ± 5,6  (10-18) |
| Phsycological interview   - no - yes | 31 (72%)  12 (28%) | 3 (100%)  - |
| Months between proposal and surgery^+^ | 6,47 ± 5,64  (1-23) | - |
| Menopausal status   - premenopausal - postmenopausal - current use of hormonal therapy for breast cancer - previous hysterectomy for benign reason | 24 (55,8 %)  12 (27,9 %)  7 (16,2 %)  - | 3 (100%)  0 (0%)  0 (0%)  - |
| Parity   - 0 - 1 - 2 - > 2 | 13 (30,2 %)  14 (32,5 %)  11 (25,5 %)  5 (11,6 %) | 0 (0%)  2 (66%)  1 (33%)  0 (0%) |
| Childbearing desire   - yes - not - unknown | 13 (30,2 %)  28 (65,1 %)  2 (4,6 %) | 2 (66 %)  1 (33 %)  - |
| Cases of cancer in I degree relatives   - none - breast cancer - ovarian cancer - breast cancer and ovarian cancer - unknown | 9 (20,9%)  15 (34,8%)  8 (18,6%)  10 (23,2%)  1 (2,3 %) | 2 (66%)  0 (0%)  1 (33%)  0 (0%)  - |
| Cases of death by cancer in I degree relatives   - none - breast cancer - ovarian cancer - breast cancer and ovarian cancer - unknown | 26 (60,4%)  6 (13,9 %)  4 (9,3 %)  0 (0%)  7 (16,2 %) | 3 (100%)  0 (0%)  0 (0%)  0 (0%)  0 (0%) |
| Personal history of breast cancer | 21 (48,8 %) | 1 (33%) |
| Risk-reducing mastectomy in personal medical history | 2 (4,6%) | 0 (0%) |

†2 patients with BRCA1 and BRCA2 mutation

‡ evaluated on 34 patients who underwent surgery
